# Metal–Organic Frameworks (MOFs) and Their Composites as Emerging Biomaterials for Osteoarthritis Treatment

**DOI:** 10.3390/biomimetics8010097

**Published:** 2023-02-27

**Authors:** Hoi-Lam Wong, Chung-Yin Tsang, Sebastian Beyer

**Affiliations:** Department of Biomedical Engineering, The Chinese University of Hong Kong, Hong Kong 999077, China

**Keywords:** Metal–Organic Frameworks, biomaterial, tissue engineering, regenerative medicine, implant, osteoarthritis, hydrogel, injectable

## Abstract

Metal-Organic Frameworks (MOFs) have emerged as a novel component in biomaterial formulations over the past 5 years. The bioactivity of MOFs in bone or cartilage tissue is mediated through the sustained delivery of metal ions, bioactive ligands, or drug molecules that are loaded into the porous MOF structures. Alternatively, bioactivity may also originate from structure-specific properties. The latter includes the availability and accessibility of open metal coordination sites for the catalytic conversion of biomolecules into active agents. This narrative highlight aims to inspire strategies to utilize MOFs for treating osteoarthritis (OA), with a special focus on augmenting hydrogel-based biomaterials with MOFs. The added value of MOFs in these hydrogel formulations is discussed, and the biological efficacy is compared to approaches applying classical injectable biomaterials for OA treatment. Possible future directions and pitfalls of these novel MOF–hydrogel composites are emphasized to assist future transition of MOFs into clinical applications.

## 1. Introduction

**Figure 1:**
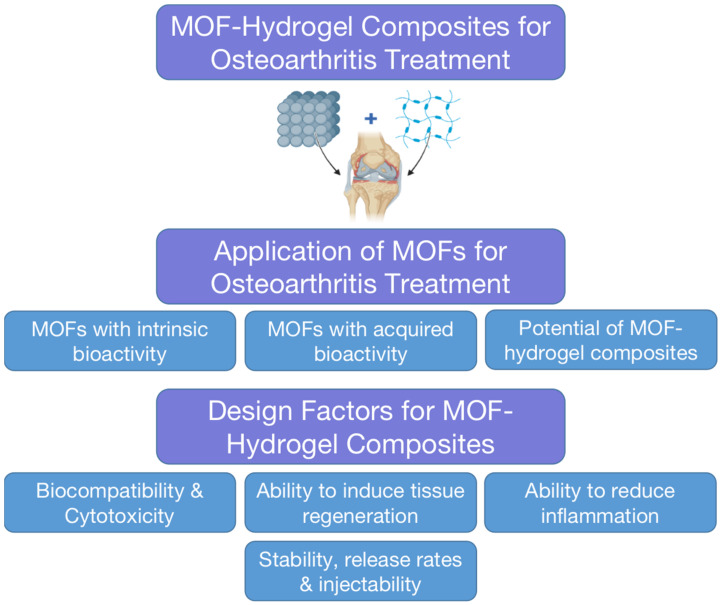
Summary of the scope and contents of this review.

Metal–Organic Frameworks (MOFs) are crystalline compounds comprising organic ligands and metal ions. The term MOF was coined in 1995 [1,2], from where this material class has advanced into one of the largest fields of contemporary materials sciences. The most facile crystallization routes for MOFs are through solution precipitation [3] or mechanochemically induced crystallization [4,5]. Notably, MOFs are often highly porous with a large inner surface area. This has inspired the investigation of small molecular weight drug delivery applications, which have been extensively reviewed elsewhere [6]. Herein, MOF-based drug delivery applications will only be discussed with new perspectives toward augmenting biomaterials or aiding OA treatment (Figure 1). MOFs may either be intrinsically bioactive or may possess acquired bioactivity. The former originates from the intrinsic properties of metal ions, ligands, or a combination of the two. The latter often involves encapsulation or augmentation with other therapeutic molecules or materials, including drugs and hydrogels. 

The intrinsic bioactivity of MOFs has previously been engineered to regulate the inflammatory response and steer wound biochemistry toward regenerative cues. In these studies, MOFs have been demonstrated to be a useful component for tissue engineering and regenerative medicine [7] and for bone-related biomaterials in particular [8]. MOFs with intrinsic bioactivity also include examples of MOFs that convert biomolecules into bioactive agents through catalytically active and accessible metal ions [9]. 

On the other hand, apart from encapsulating drugs with small molecular weight, the acquired bioactivity of MOFs through biomimetic encapsulation of biomacromolecules is an interesting research direction [10,11]. Such encapsulation, with typical examples including growth factors and nucleic acids, leads to their protection in adverse environmental conditions and their triggered release. Combining all these properties of MOFs allows for the synthesis of functional materials that can sequentially unfold their biological activity, and ultimately target different biological stages toward the regeneration of damaged tissue. 

**Figure 2:**
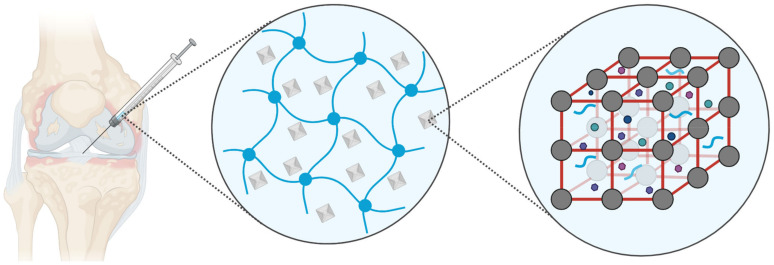
A simplified schematic showing a MOF–hydrogel composite being injected intra-articularly for OA treatment. The colloidal MOF particles may encapsulate therapeutic agents and are embedded within the hydrogel for long-term retention and sustained release applications.

While MOFs have been increasingly applied as drug delivery systems for bone-related diseases [12,13], the use of MOFs specifically for cartilage repair in the field of OA treatment has not been extensively studied. Moreover, the potential synergistic effects when augmenting MOFs with hydrogels to achieve longer retention and sustained therapeutic function remained largely unexplored until recent years. Hence, the present highlight aims to shine the spotlight specifically on MOFs as a component in biomaterial formulations for treating OA. Relevant literature has been identified through structured keyword searches using Clarivate’s Web of Science and Elsevier’s ScienceDirect databases. All works on OA involving MOFs have been curated, and special interest was directed toward studies that report injectable MOF–hydrogel composites or perspectives that may aid the future development of these composite materials (Figure 2).

### Prevalence, Pathogenesis, and Treatment Gaps of OA

Osteoarthritis (OA) is a degenerative joint disease involving disrupted homeostasis of the extracellular matrix (ECM) within the articular cartilage [14]. Such disruption results in cartilage degeneration and bone hyperplasia—the two major characteristics of OA [15]. OA has adversely affected more than 650 million patients worldwide [16] and is one of the top non-communicable diseases that leads to long years of disability. This motivates the continued quest for identifying new biomaterial compositions that ease OA conditions. OA may occur at different anatomical structures, such as the hip, and the distal phalangeal and intervertebral joints. Current studies in the field mostly focus on knee OA, which affects the entire articular knee joint, especially in the articular cartilage.

Normal articular cartilage has few chondrocytes, which are surrounded by an ECM. The cartilaginous ECM consists primarily of collagen and glycosaminoglycans among other components. The limited activity of chondrocytes, including proliferation, differentiation, and survival, is tightly regulated, specifically through crosstalk with the surrounding ECM, growth factors, cytokines, and mechanical stimuli. This tightly regulated chondrocyte activity normally maintains a healthy composition and structure of the joint [17].

Catabolic pro-inflammatory mediators are increased in OA joints, resulting in excess production of proteolytic enzymes that catalyze cartilage breakdown. Cartilage defects, in turn, amplify inflammation [18]. This ultimately results in changes in the composition or structure of the cartilage and even the subchondral bone, resulting in loss of joint integrity and function [19].

Despite decades of materials research and advancements in methods for OA treatment, two major challenges remain—the need for better materials to maximize anti-inflammatory effects without side effects and methods that facilitate effective tissue regeneration. These prevailing challenges clearly point toward intra-articular injection of drugs, proteins, genes, platelets, or cells [15]. 

To better facilitate tissue regeneration and to overcome rapid clearance of injectables from the synovial fluid, hydrogel formulations have been investigated. Many natural hydrogels such as hyaluronic acid (HA) and chitosan (CS) have high biocompatibility and increase the proliferation of chondrocytes and mesenchymal stem cells (MSCs). A future focus may be directed toward using cell-derived ECM to infer a better biological response to the host tissue [20,21]. However, even with the above advantages, tissue regeneration remains the bottleneck of OA treatment. Some problems include the inability of naturally derived hydrogels to completely replenish the functions of damaged endogenous ECM components [15]. Another challenge is the lower capacity of chondrocyte regeneration for patients over 50 years of age because of their decreased cell proliferation and ECM secretion capacity [17]. This has largely motivated experimental studies regarding the application of novel composites for OA treatment to evaluate synergies between MOFs with existing injectable biomaterials for that purpose.

## 2. MOFs for OA Treatment

**Figure 3:**
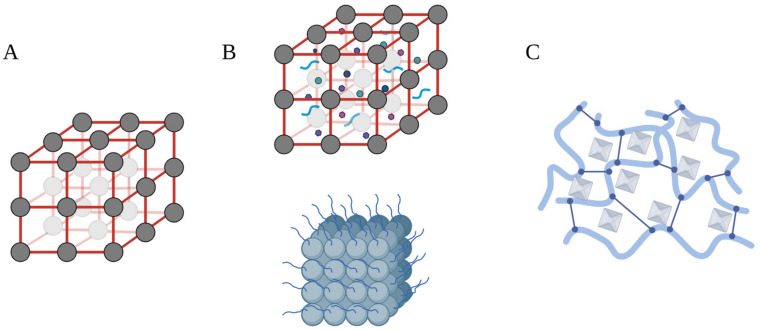
Simplified schematics of MOFs reviewed for OA treatment. (**A**) MOFs with intrinsic bioactivity. (**B**) MOFs with acquired bioactivity, which can be through encapsulation of small molecular weight drugs and biomimetic encapsulation of biomacromolecules (depicted above), along with the conjugation of therapeutic molecules (depicted below), such as peptides for targeted delivery, on the surface of MOF delivery vehicles. (**C**) MOF composite materials for OA treatment, for example, through crosslinking MOFs in hydrogels.

Intrinsic bioactivity of MOFs originates from their organic ligands, metal ions or through the 3D structure of MOFs (Figure 3). On the other hand, MOFs that act as delivery vehicles for drugs or other therapeutic molecules are considered to have acquired bioactivity.

Generally, there are two possible approaches for treating OA with biomaterials. Firstly, anti-inflammatory effects may be exerted through interception of signaling pathways. Secondly, biomaterials may deliver biologically active agents aimed at the regeneration of cartilage. While an ideal treatment may consist of both reducing inflammation and aiding tissue regeneration, most studies using MOF for OA treatment have focused on downregulating the inflammatory response. Among these articles, a very high prevalence of Zn^2+^ as the coordination center in MOFs was noted. Zinc is known to be crucial for bone homeostasis and aiding bone regeneration; hence, this may explain its use in MOFs for OA treatment [22]. Other metals that are known to have relevant bioactivity toward cartilage and bone tissue include Mg, Ca, Sr, Cu, Fe and, in particular, Co, which can be incorporated into ZIF structures [23,24]. 

### 2.1. MOFs with Intrinsic Bioactivity for OA Treatment

He and Chen identified [Zn_3_(sda)_3_(H_2_O)_2_](H_2_O)_4_, which was able to inhibit apoptosis in chondrocytes. An animal study was carried out using Sprague–Dawley (SD) rats to evaluate the efficacy of this MOF. In that study, primary chondrocytes were harvested, and DCFH-DA staining in flow cytometry revealed decreased ROS levels upon treatment with the MOF. Results from the Annexin V-FITC/PI assay have also shown the ability of the MOF to significantly downregulate chondrocyte apoptosis. Prevention of apoptosis was explained through the 3D structure of the MOF, allowing it to bind with Bcl2 [25]. Bcl2 is known to regulate and induce apoptosis in cells, and its inactivation through inhibitors is used to prevent apoptosis in general.

Another example of steering biochemistry toward a favorable healing environment was demonstrated with {[Zn(H_2_O)(H_3_L)]⋅(DMF)_2_(H_2_O)_2_}n (H_3_L = 2-(4-carboxyphenyl)-1*H*-benzo[*d*]imidazole-5-carboxylic acid, DMF = N,N-dimethylformamide). Synovial cells of OA patients have an abnormal activation of the PI3K/AKT signaling pathway. This pathway promotes inflammatory conditions and causes the production of pro-inflammatory cytokines, such as IL-18 and IL-6. In vitro studies with this Zn-based MOF have been performed on synovial cells in the logarithmic growth phase. The results obtained from the Western blot have indicated a downregulation in the PI3K/AKT signaling pathway activation level. IL-18 and IL-6 levels were also decreased, as indicated by ELISA measurements [26]. 

Other types of MOFs with intrinsic bioactivity include Mg-based MOFs such as the Mg/HCOOH system. This Mg/HCOOH system was reported to both exert anti-inflammatory effects on subchondral-related applications and to promote the regeneration of relevant bone cells. In vitro studies showed that Mg/HCOOH caused an increased proliferation of MG63 cells, which are osteoblast-like cells. In vitro RT-qPCR tests from MG63 cells also showed a downregulation in the mRNA levels of IL-1β, iNOS, and Axin2, which are three crucial inflammatory-related genes taking part in OA progression [27]. This may be attributed to the properties of the metal ion itself. Sr/HCOOH, a similar MOF to the Mg/HCOOH system, has also demonstrated the ability to regulate the expression of IL-1β, iNOS, OCN, and RANKL in qPCR results. This effect is likely attributed to the intrinsic release of Sr ions while the MOF degrades [28]. In line with the intrinsic bioactivity of MOFs mediated through their metal ion, Co-based MOFs were reported to have an anti-inflammatory effect on OA joints. Jiang et al. reported that ([Co_2_(Htpt)_2_(H_2_O)_2_]⋅CH_3_CN)_n_, H_3_tpt = polydentate 4-(2,4,6-tricarboxyphenyl)-2,2′,6,2″-terpyridine) with a 2D-layered structure and (3,3)-linked hcb network has demonstrated the ability to reduce TNF-α and IL-8 levels. Such reduction was determined through ELISA with synovial fluid obtained from OA animal models. Real-time RT-PCR was also conducted to confirm the reduction in relative expression levels of NF-κb and p53. A similar MOF, ([Co(H_2_tpt)_2_]⋅3H_2_O)), has a much lower ability to reduce the level of the inflammatory cytokines compared to ([Co_2_(Htpt)_2_(H_2_O)_2_]⋅CH_3_CN) as reported in the same study [29]. The mechanism behind the suppressive effect on the NF-κb signaling pathway was not discussed in the study. 

All the above examples have shed light on the ability to regulate inflammation with intrinsic bioactivity of MOFs, originating in their structure or individual components. 

### 2.2. MOFs with Acquired Bioactivity for OA Treatment

In contrast to MOFs with intrinsic bioactivity described in the previous section, MOFs may have acquired bioactivity, where they are used as drug delivery vehicles. This can be done through the loading of small molecular weight drugs into the porous structure of MOFs for sustained release. This acquired bioactivity may act synergistically with the intrinsic bioactivity that MOFs may have. Most studies in the biomedical field focus on ZIF-8, a polymorph of zinc(II)2-methylimidazolate, which is exceptionally easy to crystalize through solution precipitation [30]. ZIF-8 also appears to be the MOF where most studies have been conducted on the encapsulation of small molecular weight drugs and biomimetic encapsulation of biomacromolecules [10]. In the context of OA treatment, few studies report on intrinsically bioactive MOFs, while the majority of studies report on the use of MOFs as drug delivery vehicles. Of these, selected examples specifically targeting cartilage are presented in the following. 

Xue et al. reported MOF-coated carriers where the shell comprised a Fe(III)-based MOF on the surface of a mesoporous polydopamine (MPDA) core. These delivery vehicles were shown to allow for controlled, sequential dual-drug delivery of bilirubin (Br) and rapamycin (Rap), which were loaded onto the MOF shell and in the mesopores of the MPDA core, respectively. The release of both drugs can be accelerated upon pH-dependent collapsing of the MOF and near-infrared (NIR) laser irradiation. Notably, the Fe(III)-based MOF has facilitated peptide-mediated collagen-II cartilage-targeting by increasing the surface potential and surface area of the MPDA core for anchoring peptides. The MOF also enhanced the MRI imaging property of the carrier for in vivo monitoring of the therapeutic effects. Based on in vivo and in vitro studies performed on young rat models after intra-articular injection of the carrier, results have shown a successful reduction in ROS levels and enhanced autophagy activity. Results from qRT-PCR of ATDC5 cells have indicated significant downregulation in the mRNA expressions of some pro-inflammatory factors, such as TNF-α, IL-6, MMP9 and ADAMTS. On the other hand, ATDC5 cells showed an upregulation of Aggrecan, which is a major proteoglycan in the articular cartilage, and Col2a1, which accounts for type II collagen. Overall, the MOF-coated carrier has demonstrated successful downregulation of the NF-κb signaling pathway and activation of chondrocytes for effective OA treatment [31]. 

Another study by Xiong et al. reported the use of Fe-MIL-100 (MOF@HA@PCA) to deliver protocatechuic acid (PCA), a polyphenolic and hydrophilic anti-inflammatory agent. Fe-MIL-100 was chosen due to the high drug-loading capacity and pH-responsiveness. HA was incorporated to enhance the dispersibility and hydrophilicity of Fe-MIL-100, along with HA’s innate ability to reduce inflammation and provide lubrication and protection to cartilage and chondrocytes. In vitro studies of qRT-PCR, Safranin O and immunofluorescence staining on inflamed chondrocytes induced by IL-1β have shown a significant downregulation on ADAMTS5, COX-2, IL-6, iNOS, MMP-1, MMP-3, and MMP-13, indicating an effective anti-inflammatory effect and inhibition of type II collagen degradation due to PCA. In vivo studies performed on SD rats also reported attenuation of OA progression and faster cartilage regeneration after intra-articular injection of MOF@HA@PCA, as observed through macroscopic evaluation and histologic assessments of the collected joints [32]. 

Metal-phenolic networks (MPNs) appear to have not yet been investigated in the scope of tissue engineering and regenerative medicine despite their potential as naturally derived material with often known bioactivity of selected phenolic ligands [33,34]. Nevertheless, the above works have led to a future direction regarding the further exploration of targeted delivery. For example, the potential of conjugating antibodies [35] on colloidal MOF carriers to target specific tissue parts can be investigated.

On the other hand, many of the reviewed articles have focused on applying MOFs to overcome the general problem of low loading amount or lack of controlled release when using other materials for the delivery of common OA drugs, such as NSAIDs. Li et al. reported the use of Sr/PTA and Sr/HCOOH for encapsulation of ketoprofen, with Sr/PTA showing a greater loading amount of 36% as compared to only 3% for Sr/HCOOH. The ketoprofen releasing rate in these two MOFs could be best described with the Higuchi model, with a burst release in the first 8 h [28,36]. On the other hand, UiO-66, UiO-66-NH_2_, and UiO-66-NO_2_ were compared in a study regarding their loading and releasing rates of ketoprofen. Grafting -NH_2_ was shown to enhance the loading amount from 32.1% to 38%, possibly due to the affinity between the carboxylic group in ketoprofen with the -NH_2_ functional group of the framework. The functional group also extended the release time of ketoprofen, with 65% released after 24 h and almost complete release for the other two MOFs. On the contrary, grafting -NO_2_ decreased the loading amount to only 15.5% and had no effect on prolonging drug release [37]. 

Several other cyclodextrin (CD)-based MOFs (Na-γ-CD-MOF, K-γ-CD-MOF, Fe-γ-CD-MOF) that carry diclofenac sodium have been studied as well, with Fe-γ-CD-MOF showing a slightly higher entrapment efficacy of 55 ± 0.140% and gradual drug release in a period of 18 h. The cumulative drug release after 24 h was around 40% for Na-γ-CD-MOF and K-γ-CD-MOF and 60% for Fe-γ-CD-MOF. These CD-based MOFs have demonstrated prolonged drug delivery as compared to the free form of the drug [38]. These studies have demonstrated gradual advancement and have shed light on the potential directions to enhance the loading efficiency and release rate of NSAIDs. Nonetheless, a common problem of rapid initial release persists, which hinders the sustained release of the drug and remains a crucial bottleneck to be overcome in future studies.

### 2.3. MOF Composite Materials with Potential for OA Treatment

Only a few studies reported the explicit use of MOF–hydrogel composite materials specifically for OA treatment. 

Cao et al. reported the application of MOF nanozymes comprising Cu ions and tannic acid (TA) crosslinked in silk fibroin hydrogel (CuTA@SF). The composite was implanted in rabbit models with osteochondral defects for in vivo evaluation, and knee samples were collected for macroscopic and histological assessments and micro-CT observation. Results have shown the ability of the composite to induce cartilage regeneration with clear cartilage and subchondral bone structures, along with tight integration between newly formed cartilage and the adjacent host tissue. In vitro tests using bone marrow-derived mesenchymal stem cells (BMSCs) and chondrocytes from rats have also indicated that CuTA@SF yielded the most significant outcomes compared to the addition of Cu or TA alone. These outcomes include glycosaminoglycan (GAG) deposition for homeostasis of cartilage ECM, stimulating osteogenic differentiation of BMSCs, antioxidant activity, and ROS scavenging ability. This could be due to the ability of CuTA to react with ROS such as DPPH and H_2_O_2_. Under the presence of TA, SF acted as a scaffold with a smaller pore size, which was beneficial to osteochondral regeneration [39]. Overall, this study has successfully demonstrated the synergistic effects of MOF–hydrogel composites in achieving anti-inflammatory effects and promoting tissue regeneration.

Yang et al. reported the application of CD-MOF mix crosslinked in gelatin-glucosamine hydrochloride (G-GH/CL-CD-MOF@IBU), with the aim of prolonging the delivery of ibuprofen (IBU), while simultaneously providing glucosamine hydrochloride as nutrients to the surrounding chondrocytes. The composite had greater mass loss after 14 days, compared to gelatin/CL-CD-MOF@IBU and G-GH. The release rate of the composite was less steep until day 4 as compared to gelatin/CL-CD-MOF@IBU, and the cumulative release was slightly lower. The composite was seeded with MC3T3-E1 cells, and morphological observation with SEM has shown that the composite was able to provide a favorable environment for cell proliferation. Results from the CCK-8 assay also confirmed the biocompatibility of the composite [40]. The study has demonstrated the potential of synergizing MOFs with hydrogels for more prolonged delivery of drugs or nutritional supplements while exerting anti-inflammatory effects at the target area. However, no animal or clinical samples were studied.

While only a few studies on MOF–hydrogel composites have specifically addressed OA, several other works have demonstrated the use of such composites in other biomedical applications involving tissue regeneration or anti-inflammation. 

In these studies, hydrogels are incorporated due to their biological benefits, such as having high biocompatibility and water retention, promoting ECM generation, and accelerating wound healing [41]. Hydrogels could also act as the major medium for drug delivery, while selected MOFs are incorporated for prolonged and more controlled drug delivery, enhanced drug encapsulation efficiency and drug-loading ratio. These benefits could be achieved through the pH sensitivity of MOFs and their ability to encapsulate both hydrophilic and hydrophobic drugs [42]. Notably, hydrogels could also be applied as 3D-printed scaffolds for layer-by-layer assembly of hybridized MOFs for better control of drug release rates in a multi-drug delivery system [43]. These results have also shed light on the potential candidates for MOF–hydrogel composites to exert anti-inflammatory effects or induce tissue regeneration, potentially to be applied in OA treatment in future studies. Moreover, given the linkage between OA and the remodeling of neuro-muscular junctions that affect the strength of quadriceps [44], another interesting future approach may be to use electrically conductive hydrogels [45] with MOFs as composites to guide the regeneration of the neuro-muscular junction. Applying electroactive hydrogels to repair cartilage tissue with electrical stimuli [46] is another potential area to be further studied.

## 3. Factors to Consider When Designing an Ideal Composite for OA Treatment—Prospects and Potential Pitfalls

### 3.1. Biocompatibility and Cytotoxicity/Safety Assessment

Most academic research works address biocompatibility and cytotoxicity to different extents. Among many reviewed works, an attempt has been made to determine a safe dosage range (Table 1). A common method is to use methyl thiazolyl tetrazolium (MTT) assays against chondrocytes [32,37] or MG63 cells [27,28] at different MOF concentrations. The cytotoxicity was estimated by the relative cell viability compared to the untreated cells [36]. CCK-8 and live/dead assays have been applied as well. Examples of reasonably bio-safe MOFs include Mg/HCOOH [27], Sr/HCOOH, Sr/PTA [28,36], UiO-66, UiO-66-NH_2_, and UiO-66-NO_2_ [37]. These MOFs are reported to be safe at 400 mg/L, with Sr/PTA and UiO-66-based MOFs maintaining high cell viability at 800 mg/L [36,37]. In the study involving CuTA@SF, safe ranges of Cu and TA incorporated for osteochondral regeneration were 64 and 50 mg/L, respectively [39]. Two Fe-based MOFs involved in the studies were also reported to be reasonably bio-safe, including the Fe(III)-based MOF in RB@MPMW and Fe-MIL-100. Both MPMW and RB@MPMW caused no obvious cytotoxicity up to 400 mg/L [31]. Fe-MIL-100 was reported to be non-toxic until 100 μg/mL [32]. Multiple ZIF-8 composites were also reported to be safe in several studies, yet there was no clear indication regarding their exact range to achieve minimal cytotoxicity.

Nonetheless, with clinical applications of these novel materials in mind, a comprehensive pre-clinical evaluation of different MOFs and their composite materials is urgently needed. In particular, any biomaterial designed for clinical applications will need to receive regulatory approval prior to implantation into humans. 

One of the most commonly used MOFs is ZIF-8, a polymorph of Zinc(II)2-methylimidazolate. The organic ligand 2-methylimidazole is a known pharmaceutical impurity, e.g., during the synthesis of Ondansetron (an antiemetic agent). 2-Methylimidazole has also been evaluated for its carcinogenic potential by the International Agency for Research on Cancer (IARC) and was found to induce carcinoma at high doses [47]. Despite this adverse biological potential of ZIF-8, cytotoxicity and short-term biocompatibility appear to be sufficient for the intended biomedical application. Replacing ZIF-8 in some applications with an assumingly more bio-friendly ZIF-20 could point toward an interesting solution to this bottleneck [48]. 

### 3.2. Ability to Induce Tissue Regeneration

Currently, most works on MOFs for OA treatment focus on curbing inflammation as the main goal. More focus is needed to evaluate MOFs and MOF–hydrogel composites for their ability to induce tissue regeneration. Toward that end, MOFs with alkaline earth metals, such as magnesium or strontium, have been shown as potentially beneficial for OA treatment through their stimulating effect on MG63 cells or due to the known ability to inhibit chondrocyte apoptosis [28,36].

On the other hand, gelatin, for example, has been used as a scaffold for delivering growth factors due to its known property to support OA treatment [43]. Chitosan (CS) is known to induce ECM generation and the activation of macrophages to accelerate wound healing [41], which has led to its use as an injectable hydrogel for OA treatment. Easy-to-handle in-vitro assays to test the macrophage response to hydrogels are available and may even include advanced transendothelial migration assays [49,50]. This may be a worthwhile experimentation to initially assess the inherent bioactivity of MOF–hydrogel composites toward tissue regeneration. 

Steering tissue regeneration is closely related to the availability and sustained release of growth factors. Two studies have demonstrated methods to carry or deliver growth factors or stem cells sequestering growth factors with a MOF–hydrogel composite for OA treatment. 

The study by Y. Jiang et al. [43] addressed the delivery of bone morphogenic protein 2 (BMP-2), which has been encapsulated within ZIF-8 and assembled within a gelatin scaffold for implantation into the targeted tissue. This may indicate the ability to deliver growth factors that assist the regeneration of chondrocytes, such as TGF-β1, through similar methods. Another study by Fardjahromi et al. (2020) has shown the ability to use polystyrene beads that are immobilized with PDA, polyethyleneimine, and ZIF-8 for culturing hADSCs [51]. This allows the stem cells to adhere and proliferate, which may ultimately be used as a source for differentiating into chondrocytes for cartilage regeneration. Although this model was not designed specifically for stem cell delivery, similar structures or materials can be further studied to achieve this purpose.

### 3.3. Ability to Reduce Inflammation

The most direct method is to use MOFs to deliver drugs normally employed for OA with tailored release kinetics. This biomedical application of MOFs has been extensively reviewed elsewhere [6,52,53].

### 3.4. Stability and Release Rate of Composite in Different Media

The stability of a MOF–hydrogel composite is related to the release rate of bioactive ions or encapsulated drugs. In the context of OA, the stability of composites under different pH is particularly crucial, since OA joints usually have acidic pH due to cartilage degradation [32]. As reported in the articles, some common materials sensitive to acidic pH include Fe(III)-based MOFs and ZIF-8 [32,42]. Another factor affecting the stability of the composite, as reported, would be H_2_O_2_ concentrations [43]. This will also be a variable in OA joints since H_2_O_2_ is the precursor to a few other ROS [40]; hence, determining the degradation profile of composites under different H_2_O_2_ concentrations could also be significant. Nonetheless, by having materials or other small molecules [54] in the MOF composites to endow them with sensitivity toward the external environment, the release rate of therapeutic molecules can hopefully be more patient-specific in future studies.

### 3.5. Injectability

Lastly, the ideal composite should be injectable for it to be administered intra-articularly. While MOFs dispersed in solutions can be injected, hydrogel formulations may provide added values such as slowing down clearance or being liquid and only solidifying upon injection [15]. Thus, while combining MOFs with injectable hydrogels, the composite would also be able to fit the defect site after injection. The MOF–hydrogel composite materials presently known to be injectable into animal models for OA treatment include RB@MPMW [31] and MOF@HA@PCA [32]. Especially, the injectability of RB@MPMW has shed light on coated MOF microcarrier composites.

**Figure 4:**
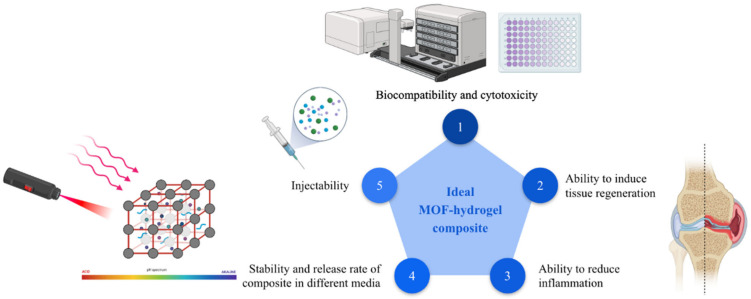
Summary of the factors to consider when designing an ideal composite, numbered in order of priority.

A graphical summary of all the factors to consider while designing and fabricating an ideal composite for OA treatment as mentioned previously can be found below (Figure 4).

## 4. Conclusions and Outlook

Treatment of OA has remained a challenge despite decades of effort toward biomaterials development. The limiting bottleneck appears to achieve tissue regeneration more than curbing inflammation, which can be achieved with established drugs and novel MOF-specific properties such as the preferential binding of cytokines. Future work may also include the sensitization or targeting of colloidal MOF carriers of an injectable hydrogel formulation to specific tissue parts through the addition of other small biomolecules. Despite an increasing number of published studies regarding MOFs in OA treatment, only a few works seem to address tissue regeneration involving guidance of in vivo stem cell differentiation toward chondrocytes. Future studies may address this particular aspect in more detail. This could be achieved using MOF–hydrogel composites for the delivery of growth factors that are biomimetically encapsulated within MOF structured or admixed to MOF–hydrogel composite materials. The intrinsic bioactivity of MOFs for assisting tissue regeneration also appears to leave much room for future investigations.

## Figures and Tables

**Table 1:** Summary of reported safe dosage ranges of bio-safe MOFs.

MOFs	Safe Dosage Range (mg/L)	Refs.
Mg/HCOOH	400	[27]
Sr/HCOOH	400	[28]
Sr/PTA	800	[36]
UiO-66, UiO-66-NH_2_, UiO-66-NO_2_	800	[37]
CuTA	64 (Cu in Cu@SF) 50 (TA in TA@SF)	[39]
MPMW, RB@MPMW	400	[31]
Fe-MIL-100	100	[32]

## Data Availability

Not applicable.
